# 

**Published:** 1895-12

**Authors:** 


### CONTENTS:

### SELECTIONS.

Article I.—The Profession and Our Dental Colleges. By Dr. Truman Brophy. - - - - - - 337
" II.—Malignant Tumors of the Mouth. - - - - 344
" III.—Disease of the Antrum of Highmore from a Medical Standpoint. - - - - - - - - 349
" IV.—Fracture of the Superior Maxilla. By W. D. M. Mason. - - - - - - - - 357
" V.—Occlusion and Mastication. By W. H. H. Barker. 358
" VI.—The Tincture of The Chloride of Iron. By G. S. Martin, L. D. S., D. D. S. - - - - - 360
" VII.—National Association of Dental Faculties - - 364
" VIII.—Cleft Palates—Cases in Practice. By Dr. Benjamin Simons. - - - - - - - - 369
" IX.—Dental Jurisprudence.—Infection. By H. R. Wiley, A. B., - - - - - - - - - 373

### OBITUARY.

Dr. Thomas H. Chandler. - - - - - - 375
Sir John Tomes. - - - - - - - - - 377

### EDITORIAL, ETC.

The Care of Children's Teeth. - - - - - - - 380

### MONTHLY SUMMARY.

Disturbance Caused by Diseases of the Teeth. - - - - 383
Abscesses. - - - - - - - - - - 384
To Open Up a Blind Abscess. - - - - - - - 384

